# Kinematic Assessment to Measure Change in Impairment during Active and Active-Assisted Type of Robotic Rehabilitation for Patients with Stroke

**DOI:** 10.3390/s21217055

**Published:** 2021-10-25

**Authors:** Donghwan Hwang, Joon-Ho Shin, Suncheol Kwon

**Affiliations:** 1 Department of Rehabilitation & Assistive Technology, National Rehabilitation Center, Ministry of Health and Welfare, Seoul 01022, Korea; dh0746@korea.kr or; 2 Translational Research Program for Rehabilitation Robots, National Rehabilitation Center, Ministry of Health and Welfare, Seoul 01022, Korea; 3 Department of Neurorehabilitation, National Rehabilitation Center, Ministry of Health and Welfare, Seoul 01022, Korea

**Keywords:** stroke, robotic rehabilitation, upper extremity, kinematics features, Fugl-Meyer

## Abstract

Analysis of kinematic features related to clinical assessment scales may qualitatively improve the evaluation of upper extremity movements of stroke patients. We aimed to investigate kinematic features that could correlate the change in the Fugl-Meyer Assessment (FMA) score of stroke survivors through upper extremity robotic rehabilitation. We also analyzed whether changes in kinematic features by active and active-assisted robotic rehabilitation correlated differently with changes in FMA scores. Fifteen stroke patients participated in the upper extremity robotic rehabilitation program, and nine kinematic features were calculated from reach tasks for assessment. Simple and multiple linear regression analyses were used to characterize correlations. Features representing movement speed were associated with changes in FMA scores for the group that used an active rehabilitation robot. In contrast, in the group that used an active-assisted rehabilitation robot, features representing movement smoothness were associated with changes in the FMA score. These estimates can be an important basis for kinematic analysis to complement clinical scales.

## 1. Introduction

Approximately 30–60% of stroke survivors suffer from upper extremity dysfunction [1]. They experience limited activities of daily living (ADLs) and social participation [1,2]. During stroke rehabilitation, repetitive and intense treatment tasks are important factors to promote neuroplasticity and improve functional outcomes [3,4]. Robotic rehabilitation can encourage active participation with highly repetitive tasks that interact with specific motor functions [5]. Recently, the use of robots for upper extremity rehabilitation has increased to provide high-intensity rehabilitation training [2,3]. According to a recent systematic review, the beneficial effects of robotic rehabilitation on motor function recovery for upper extremity dysfunction in stroke patients have been established [6,7,8]. Moreover, some reports suggest that the active robotic device (ACT) can be used to evaluate the therapeutic effect on motor learning by analyzing the post-stroke patients’ kinematics for motor and functional impairment [9]. However, its effectiveness remains controversial [10,11].

Rehabilitation robots that users actively use can be classified as active and active-assistive robot devices according to the type of manipulation. ACT provides only gravity compensation power for the user’s rehabilitation body segments and the robot [12]. ACT should be operated by the user during the training, whereas the active assistive robotic device (ACAS) actively aids in the user’s movement. ACAS provides assistive force for the user’s movement and helps the user to reach a target point even if he/she is not able to move at all [13,14,15]. The provision of active assisted force of the robot may benefit patients who struggle with spontaneous movement, as the robot can be trained for a desired path or speed. Conversely, an active robot could induce voluntary participation in the patient, thereby unleashing the patient’s motor potential.

Clinical scales [16], such as the Fugl-Meyer Assessment (FMA) [17] and Wolf Motor Function Test (WMFT) are frequently used to assess motor functionality in patients after stroke. However, clinical scales are not sensitive enough to capture the quality of sensory and motor performance. Kinematic assessments can increasingly be used as motor outcome measures during upper extremity robot-assisted training, in addition to clinical scales [18]. Therefore, it is necessary to use kinematic features for the sensitive and objective evaluations of patients with post-stroke dysfunction [19,20]. Previous studies on robotic rehabilitation and clinical assessments have already identified the relationship between FMA scores and kinematic features in stroke patients. Lee et al. and Bertani et al. considered changes in the clinical variables of different robotic rehabilitation systems but did not consider various kinematic features [21,22]. Since robotic rehabilitation has a characteristic of heterogeneity, it may be helpful to analyze the properties according to the type of each robot, as done in this study.

We hypothesized that rehabilitation using two different robots would result in different kinematic changes. Recently, we demonstrated discrepant changes in kinematics between ACT and ACAS, although clinical measurements, including FMA and WMFT, did not find any difference [15]. The purpose of this study was to determine whether the kinematic features that cause changes in FMA differ during rehabilitation with ACT and ACAS robotic rehabilitation. Therefore, we performed a regression analysis of the change in FMA, a clinical assessment indicating impairment using changes in the kinematic data of a patient’s reaching task during rehabilitation.

## 2. Materials and Methods

### 2.1. Participants

This study enrolled 20 patients with upper extremity dysfunction due to stroke who were admitted to the National Rehabilitation Center, Republic of Korea, between March 2017 and December 2017. The study was approved by the institutional review board of the Rehabilitation Hospital (approval no. NRC-2017-01-007). Informed consent was obtained from all the subjects involved in the study. The inclusion criteria were as follows: (1) 19 years of age or older, (2) the presence of hemiplegia due to ischemic or hemorrhagic stroke, (3) stroke duration greater than 3 months, (4) hemiplegic shoulder and elbow flexion/extension with a score of 3 or 4 based on the Medical Research Council scale for strength, (5) a Fugl-Meyer assessment score (FMA) of 21–50 for the affected upper extremity, (6) shoulder and elbow flexor spasticity with a modified Ashworth scale score of ≤1+, (7) cognitive function sufficient to understand and obey the protocol of this study, and (8) the absence of limits in the range of motion of the shoulder and elbow joints as determined by the neutral zero method [23]. The exclusion criteria were as follows: (1) a history of surgical treatment of the affected upper extremity; (2) upper extremity musculoskeletal problems, such as fracture, contracture, and shoulder subluxation of more than two finger breadths; and (3) cybersickness, that is, nausea or vomiting while viewing the screen.

### 2.2. Study Design

Participants were randomly divided into the ACT and ACAS intervention groups. Each participant received 20 sessions of robotic rehabilitation (five times a week for 4 weeks). Participants were trained in a game-based virtual reality environment that focused on proximal upper limb movements provided by Hocoma Inc. Each treatment lasted for 30 min. We obtained FMA and kinematic features at baseline before intervention (T0), immediately after intervention at two weeks (T1), and at four weeks (T2). The participants performed reaching tasks to generate three-dimensional trajectory data used for calculating the features. Specific details on rehabilitation training are described by Park et al. [15].

To compare the correlation between changes in the FMA through upper extremity rehabilitation of active and active-assistive rehabilitation robots and changes in kinematic features, the active rehabilitation robot Armeo^®^Spring (Hocoma Inc., Zurich, Switzerland) was used, and the active-assistive rehabilitation robot used Armeo^®^Power (Hocoma Inc., Zurich, Switzerland). The ACT group used the exoskeleton-activated robot Armeo^®^Spring for three-dimensional upper extremity rehabilitation. The Armeo^®^Spring provides only the force to counteract the gravity of the upper extremity and the robot using a spring and not a robot actuator. The ACAS group used the Armeo^®^Power, which is also an exoskeleton-activated robot for three-dimensional upper extremity rehabilitation. Armeo^®^Power provides force through the actuator to support the affected arm movement according to a set range. Participants were trained on the same virtual reality environment as those included in the ACT group.

At the first visit, each patient was classified into ACT or ACAS groups, and the robotic device was adjusted so as not to restrict the movement of the patient’s upper extremities in a three-dimensional workspace. Each patient underwent the prepared robotic rehabilitation training program. During the subsequent visits, the patient was placed on the device in the same manner and performed the same exercises.

### 2.3. Outcome Measure

We obtained the FMA and the kinematic outcomes. Outcome measures were checked at baseline (T0) as well as two (T1) and four weeks after the intervention (T2).

#### 2.3.1. Clinical Assessment

We evaluated the FMA score to measure motor impairment according to the International Classification of Functioning, Disability, and Health (ICF) concept [24]. We assessed the FMA score, which is a quantitative indicator of movement impairment after stroke A higher FMA score indicates a lower impairment [25]. We used FMA-UE (shoulder, elbow, forearm, wrist, and hand; 33 items, 0–66) and FMA-prox (shoulder, elbow, and forearm; 18 items, 0–36) to confirm upper extremity dysfunction.

#### 2.3.2. Kinematic Assessment

**Figure 1:**
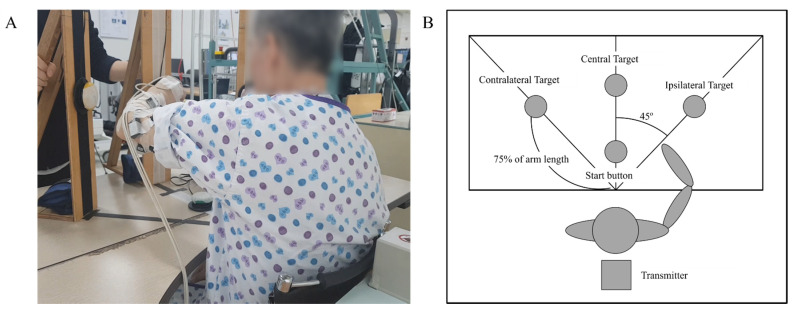
(**A**) A participant performing the reaching task. (**B**) Point-to-point reaching task evaluation table configuration.

**Figure 2:**
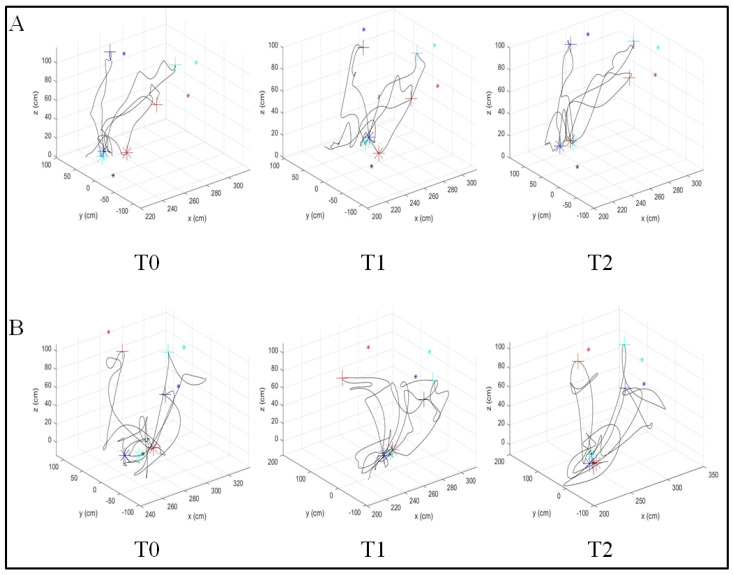
Example of 3D trajectory according to the reaching task of stroke patients in (**A**) the ACT group and (**B**) the ACAS group. This plot represents the actual trajectory in reaching the target. The asterisk represents the actual position of the target, the cross represents the hand position when the patient reaches the target. The red, cyan, and blue colors represent the medial, middle, and lateral directions, respectively.

The kinematic features were evaluated using the three-dimensional trajectory of the reaching task. The patient performed a reaching task toward three targets (Figure 1A) in the lateral, middle, and medial orientations (Figure 1B) at a distance equal to 75% of the patient’s arm length. The task was performed three times in one trial. The reach task sequence was as follows: (1) start point (on table), (2) lateral target, (3) start point, (4) middle target, (5) start point, (6) medial target, and (7) start point. The patient clicked the button at the starting and target points. The movement between the two coordinates of the start and target points was defined as a single movement. Upper extremity trajectories were recorded to quantify detailed information about motor impairment. The movement was captured using trakSTAR^TM^ (Ascension Technology Corp, Street Louis, MO, USA), which records three-dimensional coordinates at a frame rate of 200 Hz (Figure 2). It was recorded by attaching a sensor to the tip of the index finger. The collected data were filtered using a 6 Hz second-order Butterworth low-pass filter.

In this study, nine kinematic features were calculated with reference to previous studies, as listed in Table 1. The filtered trajectory data were calculated as kinematic features through custom code using MATLAB (R2019b, Mathworks Inc, Natick, MA, USA). The features can be classified into five types in terms of movement speed, efficiency, accuracy, smoothness, and control strategy.

### 2.4. Data Analysis

Statistical analyses were performed using the SPSS software (version 20.0, IBM Corp, Armonk, NY, USA). The significance threshold for the *p*-value was set to 0.05. Repeat measures of analysis of variance (RM-ANOVA) for group (ACAS and ACT) and time (T0, T1, or T2) were performed to compare the effect of each intervention over time and to evaluate time × group interactions with a post hoc Tukey test. When the sphericity assumption was not satisfied, the Greenhouse–Geiser correction was applied [32]. Wilcoxon signed-rank tests were performed for intergroup comparisons of kinematic features. Outliers were detected and removed from statistical analysis using Tukey’s method [33].

Simple linear regression was performed (with changes in each kinematic feature) on the change in FMA. Then, multivariable linear regression was performed (with changes in the kinematic features) on the change in FMA. Proper features for multivariable linear regression were selected using the forward method [34]. Model assumptions were validated through residual analysis, coefficient of variance inflation (VIF), and predicted probability plots. We selected features for the regression model when VIF was less than two, and the predicted probability plots of the standardized residuals were found to be close to the normal values for all regression models.

## 3. Results

Of the 20 participants, one participant from the ACT group dropped out due to transfer to another hospital. Two participants from the ACT group and two from the ACAS group were excluded from the analysis because of signal loss in some sessions during the evaluation; thus, 15 participants (seven in the ACT group, eight in the ACAS group) completed all four assessment sessions (Table 2).

### 3.1. Comparison of Clinical Assessment

There was a significant effect of time on both FMA-UE (*p* = 0.025) and FMA-prox (*p* = 0.003) without the time × group interaction on FMA-UE (*p* = 0.907) or FMA-prox (*p* = 0.921) (Table 3).

### 3.2. Comparison of Kinematic Assessment

There was a significant effect of time on MaxSp (*p* = 0.001), MeanSp (*p* < 0.001), RMMS (*p* = 0.004), and SPARC (*p* = 0.001) in the comparison of the ACT and ACAS groups (Table 3). The movement speed indicated that there was a significant effect of time × group interaction. The ACAS group showed a better improvement than the ACT group with regard to MaxSp from 0 to 4 weeks (*p* = 0.018) and from 2 to 4 weeks (*p* = 0.028), with regard to MeanSp from 2 to 4 weeks (*p* = 0.018) and TPeakSp from 2 to 4 weeks (*p* = 0.018). In contrast, the ACT group exhibited better RMMS progression than the ACAS group from 0 to 4 weeks (*p* = 0.018) and from 2 to 4 weeks (*p* = 0.028).

### 3.3. Regression Performance

In the ACT group, the change in FMA-UE could be explained by MaxSp (*p* < 0.001), MeanSp (*p* < 0.001), HPR (*p* < 0.001), TEr (*p* < 0.001), MAPR (*p* = 0.002), and TPeakSp (*p* = 0.002). In the ACAS group, the change in FMA-UE could be explained by MaxSp (*p* < 0.001), TEr (*p* < 0.001), RMMS (*p* < 0.001), MAPR (*p* < 0.001), and TPeakSp (*p* < 0.001). The results of the simple linear regression analysis of the kinematic features against FMA-UE and FMA-prox are listed in Table 4 and Table 5.

Multiple regression analysis models for FMA-UE and FMA-prox of the ACT and ACAS groups were derived. For the ACT group, the combined TEr and MaxSp regression model estimated the change in FMA-UE (adjusted R^2^ = 0.648), and the combined MeanSp, MAPR, TEr, and HPR regression models showed higher estimates of change in FMA-prox (adjusted R^2^ = 0.677). For the ACAS group, TE, MAPR, TPeakSp, RMMS, SPARC, and MaxSp combined regression models estimated changes in FMA-UE (adjusted R^2^ = 0.599), and the combined TEr, TPeakSp, MAPR, and RMMS regression models yielded lower estimates of change in FMA-prox (adjusted R^2^ = 0.542). The results of the multiple linear regression analysis of the kinematic features against FMA-UE and FMA-prox are listed in Table 6 and Table 7.

## 4. Discussion

We demonstrated discrepancies in the kinematic estimates of impairment changes for the two types of rehabilitation robots. In the ACT group, significant changes were confirmed pre- and post-rehabilitation of MeanSp, RMMS, and SPARC. In the ACAS group, significant changes were confirmed pre- and post-rehabilitation of MaxSp, MeanSp, and SPARC. However, we did not confirm any significant pre- and post-rehabilitation change in FMA-UE and FMA-prox. Many studies using the reaching task of robotic rehabilitation have used FMA as an evaluation tool. However, it can be suggested that FMA is an insufficient evaluation tool to detect changes in patient’s motor function owing to the reaching task of robotic rehabilitation [35].

TEr and MaxSp were correlated to upper extremity impairment in the ACT group and could explain the FMA-UE score (adjusted R^2^ = 0.648). The MeanSp, MAPR, TEr, and HPR were related to FMA-prox and could explain it (adjusted R^2^ = 0.677) SPARC, TPeakSp, and MaxSp were related to FMA-UE in the ACAS group and could explain it (adjusted R^2^ = 0.599). TEr, TPeakSp, MAPR, and RMMS were related to FMA-prox and could explain it (adjusted R^2^ = 0.542). The kinematic features that account for the FMA are different between the two robot groups. In other words, it can be said that the cause of the motor function recovery due to the ACT and ACAS is different. The ACT group seems to be accelerated by voluntary training to perform the task on its own, and the ACAS group had a faster TPeakSp (Table 3), which can be said to have improved the continuous and uninterrupted control strategy when reaching the target by the assistive force of the robot [36,37,38].

Additionally, active and active-assisted robotic rehabilitation are manifested as improvements in different kinematic characteristics. In both types of rehabilitation, speed, accuracy, and smoothness could explain the changes in FMA and FMA-prox. The control strategy highly correlated the change in FMA in the ACAS group (Table 6 and Table 7), and the training to correct the trajectory during rehabilitation could be affected by the intervention of the active supporting force of the robot. In addition, the kinematic features of movement efficiency were highly correlated with changes in FMA in the ACT group, which may be affected by the patient’s voluntary-effort-induced movement. The movement speed was related to the FMA of the ACT group, and the motion control strategy was related to the change in the FMA of the ACAS group, which was consistent with the results presented in this study. Kinematic features showed higher correlation in the ACT group compared to that in the ACAS group, and changes in the FMA-UE and FMA-prox scores were slightly better explained in the ACT group. This suggests that the change in FMA in the ACT group with voluntary rehabilitation motivation could have a higher correlation with the change in kinematic features.

In a previous study, the changes in clinical scale were estimated by varying the kinematic characteristics by providing ACAS. In particular, it was shown that the number of velocity peaks, a feature indicating smoothness, was correlated with FMA-UE (adjusted R^2^ = 0.625) [39]. These results are partly consistent with those of the ACAS group. However, rehabilitation was provided by only the InMotion2 robot (Interactive Motion Technologies, Inc.), which is an end effector rehabilitation robot that achieves two-dimensional motion. A comparison of the ACT group was not made. Despite not considering various kinematic features, its high estimation could be attributed to the limited movement in the planar movement. Hussain et al. confirmed that kinematic features calculated from the point-to-point reaching task were related to FMA-UE and the action research arm test (ARAT) in end-effector robot rehabilitation with the use of a virtual environment. They suggested that clinical evaluation can be improved using kinematic features, as understanding the association between clinical and kinematic features could identify factors affecting movement impairment and activity limitations [40]. Movement speed and smoothness were confirmed to correlate with the FMA. In addition, Park et al. confirmed that the change in smoothness was correlated with the change in FMA in upper extremity active robot rehabilitation [41], and the results were similar to our ACT group results. However, the task to reach the goal was performed in a small workspace that did not induce shoulder movement, which explains its low correlation with FMA. It can be said that voluntary movement with ACT improves movement quality, such as speed and efficiency, and assisted movement with ACAS limits the patient’s incorrect movement path and improves movement quality, such as smoothness and control strategy. In the ACT group, we assumed that movement impairment could be estimated by kinematic features, because assistive forces are not provided during robotic rehabilitation; however, in the ACAS group, because assistive forces are provided, kinetic data may be needed to explain movement impairment. In the ACT group, FMA-prox demonstrated a higher correlation with the smoothness index than that of FMA-UE, which was consistent with the results of a previous study [41]. These results suggest that the rehabilitation of the ACT group could be related to shoulder movement [40], and the proximal movement could have been more active than the distal movement during rehabilitation. Smoothness can be a good indicator of impairment recovery [42]. In contrast, in the ACAS group, FMA-UE showed a higher correlation with smoothness. This could indicate that the distal and proximal parts of the upper extremity were activated and moved together in a desired stretching way with the degree of freedom of the joint constrained by the robot; however, additional research is needed to confirm this hypothesis.

The results of our study indicate the relationship of FMA, a clinical scale of impairments, with nine kinematic features of five characteristics. In particular, we confirmed that the change in FMA in the ACT group demonstrated a correlation with MaxSp, TEr, and MAPR, and the change in FMA in the ACAS group demonstrated a correlation with MaxSp, TEr, RMMS, MAPR, SPARC, and TPeakSp. These results indicate that FMA can be estimated from kinematic features, thereby providing a basis for standardization as an index to complement the clinical scales.

## 5. Conclusions

Our findings suggest that changes in different kinematic features using active and active-assisted robotic rehabilitation correlated with changes in FMA-UE and FMA-prox, which is an impairment indicator. Thus, the kinematic features of active and active-assisted robotic rehabilitation can be used to objectively and quantitatively assess upper extremity dysfunction. However, these results confirm correlation, and not causation. Further studies are needed to establish a causal relationship. Common features such as movement speed, accuracy, and smoothness can estimate the change in upper extremity impairment during both active and active-assistive robotic rehabilitation, whereas control strategy and efficiency can be estimated using active robotic rehabilitation and active assistive robotic rehabilitation, respectively. Nevertheless, these kinematic features can only explain part of the upper extremity impairment. Kinetic data may be needed as a complementary means to indicate impairment, and we intend to analyze the relationship between kinetic data and clinical scales of impairment. In addition, to use the kinematic feature as an index to complement the clinical scales, further research on the estimation of the other clinical scales of impairment as the kinematic features must be performed.

## Figures and Tables

**Table 1:** Descriptions of kinematic features.

Kinematic Features	Abbreviation	Unit	Description	Characteristic
Max Speed	MaxSp	mm/s	the fastest speed in each speed profile during each movement the higher values are related to a faster movement	Movement Speed
Mean Speed	MeanSp	mm/s	the average of the motion speeds of each speed profile during each movement the higher values are related to a faster movement	
Hand Path Ratio	HPR	dimensionless	the ratio between the actual distance to the desired distance in a single movement between the starting point and the target point [26,27] the higher values are related to a longer hand trajectory during the movement.	Movement Efficiency
Movement Deviation	MD	mm	the mean absolute value of the vertical distance from the theoretical path to each point on the real path [28] ※ the lower values indicate that the real path is similar to the desired path	Movement Accuracy
Target Error	TEr	mm	the minimum distance from the index finger to the target location at the end of the movement as intended by the subject [26] ※ the lower values indicate that the target has been reached	
Ration between Mean and Maximum Speed	RMMS	%	the ratio of the mean to the maximum speed the value of a healthy subject should be close to one	Movement Smoothness
Mean Arrest Period Ratio	MAPR	%	the ratio of the movement time in which the hand stopped to the total movement time. The arrested hand was defined to have less than 20% of the average speed during each movement [29] the lower values are related to a more continuous movement	
SPARC	SPARC	dimensionless	the amplitude and Fourier magnitude spectrum from the velocity profile of the hand movement [30] the lower values indicate that there is more sub-movement in the movement	
Time to Peak Speed	TPeakSp	s	the time from the start of the movement to the maximum speed during the movement [31] the lower values indicate that the maximum speed of movement is reached faster	Movement Control Strategy

**Table 2:** Baseline characteristics of the participants.

Demographic Data and Clinical Characteristics	ACT Group (n = 7)	ACAS Group (n = 8)
Age	51.11 ± 14.85	53.60 ± 11.23
Time after stroke onset (months)	9.38 ± 5.79	9.60 ± 5.79
Stroke type (infarction/hemorrhage)	3/4	3/5
Hemiplegic side, right	4	4
Sex, male	6	6
FMA-prox	22.71 ± 5.94	21.00 ± 4.96
FMA-UE	31.29 ± 10.00	28.63 ± 11.44

**Table 3:** Comparison of performance between the ACT and ACAS groups at T0, T1, and T2.

Variable	ACT Group (n = 7)			ACAS Group (n = 8)			Time		Time × Group	
	T0	T1	T2	T0	T1	T2	F	*p*-Value	F	*p*-Value
FMA-UE	31.30 ± 10.00	34.10 ± 11.60	35.30 ± 9.00	24.70 ± 3.10	27.40 ± 4.80	27.70 ± 2.10	4.383	0.025	0.094	0.907
FMA-prox	22.70 ± 5.90	24.30 ± 6.80	26.00 ± 6.00	19.40 ± 2.40	21.00 ± 3.00	22.30 ± 1.90	8.475	0.003	0.055	0.921
MaxSp (mm/s)	3.55 ± 1.60	3.85 ± 1.92	3.59 ± 1.50	2.94 ± 1.03	3.51 ± 0.87	4.25 ± 1.07 ^†,‡^	9.369	0.001	9.163	0.001
MeanSp (mm/s)	1.13 ± 0.60	1.41 ± 0.78	1.46 ± 0.63 ^†^	1.06 ± 0.51	1.21 ± 0.34	1.62 ± 0.46 ^†,‡^	33.842	<0.001	5.516	0.022
HPR	0.92 ± 0.23	0.89 ± 0.25	0.95 ± 0.16	0.93 ± 0.15	0.87 ± 0.13	0.88 ± 0.10	0.916	0.407	0.646	0.521
MD (mm)	81.61 ± 7.20	76.84 ± 20.69	71.29 ± 19.46	80.80 ± 21.96	77.61 ± 11.91	76.43 ± 13.58	1.956	0.188	0.741	0.499
TEr (mm)	30.72 ± 14.83	29.56 ± 18.21	30.89 ± 20.59	37.22 ± 12.77	33.14 ± 10.70	28.42 ± 7.30	1.1013	0.364	1.120	0.343
RMMS (%)	0.31 ± 0.05	0.35 ± 0.08	0.41 ± 0.08 ^†,‡^	0.36 ± 0.06	0.36 ± 0.06	0.39 ± 0.04	7.793	0.004	2.340	0.128
MAPR (%)	0.10 ± 0.06	0.10 ± 0.07	0.09 ± 0.07	0.08 ± 0.04	0.07 ± 0.04	0.07 ± 0.04	0.721	0.475	0.440	0.615
SPARC	−8.57 ± 1.01	−8.63 ± 1.88	−7.26 ± 1.06 ^†^	−9.91 ± 2.21	−8.84 ± 1.87	−7.91 ± 1.70 ^†,‡^	15.177	0.001	0.371	0.699
TPeakSp (s)	0.60 ± 0.22	0.60 ± 0.23	0.53 ± 0.23	0.67 ± 0.25	0.59 ± 0.21	0.50 ± 0.16 ^‡^	2.125	0.154	0.398	0.630

Values are presented as mean ± standard deviation. ACT, active; ACAS, active-assistive; FMA-UE, Fugl-Meyer assessment upper extremity; FMA-prox: Fugl-Meyer assessment proximal upper extremity; MaxSp: maximum speed; MeanSp: mean speed; HPR: hand path ratio; MD: movement detection; TEr: target error; RMMS: ratio of mean and maximum speeds; MAPR: mean arrest period ratio; SPARC, TPeakSp: time-to-peak speed. ^†^ Wilcoxon signed-rank test t0–t2 (*p* < 0.05); ^‡^ Wilcoxon signed-rank test t1–t2 (*p* < 0.05).

**Table 4:** Comparison of simple linear regression analysis of independent kinematic features for FMA-UE in the ACT and ACAS groups.

	ACT Group (n = 7)			ACAS Group (n = 8)		
Independent Variables	Unstandardized B	Adjusted R^2^	*p*-Value	Unstandardized B	Adjusted R^2^	*p*-Value
MaxSp	3.664	0.337	<0.001	3.278	0.173	<0.001
MeanSp	8.232	0.290	<0.001	0.638	−0.005	0.721
HPR	−21.655	0.166	<0.001	−7.203	0.008	0.119
MD	0.008	−0.006	0.800	0.016	−0.004	0.612
TEr	−0.494	0.522	<0.001	−0.538	0.364	<0.001
RMMS	12.629	0.011	0.104	−39.030	0.134	<0.001
MAPR	22.386	0.058	0.002	59.103	0.228	<0.001
SPARC	0.430	0.008	0.136	0.066	−0.006	0.832
TPeakSp	−6.459	0.058	0.002	−10.652	0.123	<0.001

**Table 5:** Comparison of simple linear regression analysis of independent kinematic features for FMA-prox in the ACT and ACAS groups.

	ACT Group (n = 7)			ACAS Group (n = 8)		
Independent Variables	Unstandardized B	Adjusted R^2^	*p*-Value	Unstandardized B	Adjusted R^2^	*p*-Value
MaxSp	2.486	0.495	<0.001	1.106	0.124	<0.001
MeanSp	6.126	0.514	<0.001	−0.039	−0.006	0.956
HPR	−12.488	0.176	<0.001	−2.099	0.002	0.247
MD	0.001	−0.007	0.973	−0.003	−0.005	0.805
TEr	−0.200	0.268	<0.001	−0.204	0.340	<0.001
RMMS	12.345	0.046	0.004	−13.161	0.098	<0.001
MAPR	9.107	0.027	0.023	20.227	0.172	<0.001
SPARC	0.226	0.006	0.164	0.111	−0.001	0.834
TPeakSp	−3.425	0.051	0.003	−4.796	0.164	<0.001

**Table 6:** Multivariable linear regression analysis of kinematic features for FMA-UE in the ACT and ACAS groups.

	ACT Group (n = 7)					ACAS Group (n = 8)			
Kinematic Characteristic	Selected Variables	Unstandardized B	Adjusted R^2^	*p*-Value	Kinematic Characteristic	Selected Variables	Unstandardized B	Adjusted R^2^	*p*-Value
Speed	MaxSp	2.384	0.648	<0.001	Speed	MaxSp	1.107	0.599	<0.001
Efficiency	-	-			Efficiency	-	-		
Accuracy	TEr	−0.405			Accuracy	TEr	−0.360		
Smoothness	-	-			Smoothness	RMMS	−14.479		
						MAPR	35.345		
						SPARC	−0.532		
Control strategy	-	-			Control strategy	TPeakSp	−8.889		

**Table 7:** Multivariable linear regression analysis of kinematic features for FMA-prox in the ACT and ACAS groups.

	ACT Group (n = 7)					ACAS Group (n = 8)			
Kinematic Characteristic	Selected Variables	Unstandardized B	Adjusted R^2^	*p*-Value	Kinematic Characteristic	Selected Variables	Unstandardized B	Adjusted R^2^	*p*-Value
Speed	MeanSp	5.663	0.677	<0.001	Speed	-	-	0.542	<0.001
Efficiency	HPR	−3.551			Efficiency	-	-		
Accuracy	TEr	−0.073			Accuracy	TEr	−0.127		
Smoothness	MAPR	12.874			Smoothness	RMMS	−8.773		
						MAPR	11.550		
Control strategy					Control strategy	TPeakSp	−1.173		

## Data Availability

The data presented in this study are not available because of reasons concerning the privacy of the subjects.
